# Osteotomy treatments and post-operative fixations for Blount disease: A systematic review

**DOI:** 10.1016/j.amsu.2022.103784

**Published:** 2022-05-21

**Authors:** Panji Sananta, Joko Santoso, Muhammad Alwy Sugiarto

**Affiliations:** Orthopaedics and Traumatology Department, Faculty of Medicine, Universitas Brawijaya-RSUD Dr. Saiful Anwar, Malang, East Java, Indonesia

**Keywords:** Blount disease, Tibia vara, Osteotomy, Fixation

## Abstract

**Background:**

Blount disease is a developmental abnormality characterized by abnormal ossification of proximal tibia, resulting in lower limb deformities with tibia vara. The condition worsens into knee deformity, gait abnormalities, and premature medial compartment osteoarthritis if left untreated. Managements of those deformities have also advanced in line with the understanding of the deformities. Without proper care management, they could lead into residual and translational deformities, increase of recurrence, and complicate the revision surgery.

**Methods:**

This study aims to enrich our understanding about the recent advances of treatments for Blount disease by reviewing 15 articles published with osteotomy surgeries and fixation methods. We also highlight many aspects of pre-operative assessment and planning, post-operative complications and recurrence, patients' follow-up, and overall satisfaction from patients’ self-assessment.

**Results:**

The scope of this review is considered small but still covers various efforts to manage Blount diseases, including single-stage double osteotomy, grafting fibular fragments into tibia, two comparison studies, two unique case study, and experimental techniques to manage special cases requiring novel procedures.

**Conclusion:**

Careful surgical planning, acute or gradual correction options, and the use of fixator should be tailored to individual cases.

## Highlights

• Blount disease management is preferable before age four years old.
• Osteotomy surgeries and fixation methods is recommended for Blount disease.
• Mostly satisfactory outcome was reported using this methods.

## 1 Introduction

Blount disease is an idiopathic developmental abnormality characterized by disordered endochondral ossification of medial proximal tibial physis, resulting in a multiplanar lower limb deformities with pronounced tibia vara [1]. In 1937, Blount characterized the infantile tibia vara (ITV) that is apparent before age four, and the late onset tibia vara (LOTV) that develops in adolescents before skeletal maturity [2]. While Blount implied that deformity occurs solely in the frontal plane, it is now believed that other deformities like proximal tibial procurvatum and internal torsion are among the other common deformities associated with Blount disease. Managements of those deformities have also advanced. If deformities were being overlooked during the management of the disease, they could lead into residual and iatrogenic translational deformities, increase the incidence of recurrence, and complicate the revision surgery.

The usual procedures to treat Blount disease are technically demanding and complicated. This leads to longer healing period and lower patient compliance [4]. Blount disease has been long studied within numerous reviews and reports, but the method of correction and fixation remains debatable. Gradual distraction osteogenesis is generally seen as better practice to manage Blount disease as it is believed to be safer and more accurate to deal with multiplanar deformities, even limb length differences [1]. However, acute correction of angular and rotational deformity provides a more practical strategy and shorter, easier monitoring [1]. For late onset and severe deformities, proximal tibial osteotomy for correction regardless of the fixation method was generally suggested. However, there is few evidence to prescribe one form of fixation over the other [5].

## 2 Methods

All methods in this study followed the Preferred Reporting Items for Systematic Reviews and Meta-Analyses for Network Meta-Analyses (PRISMA) [3]. and used AMSTAR (Assessing the methodological quality of systematic reviews) [6]. This study also has been registered in PROSPERO with number ID: 316258.

### 2.1 Study selection

In initially searching methods, we use only Research article such as Prospective, retrospective, case series, and case reports were included in the study. Studies must be written in English and were published within the last 10 years. Books, reviews, editorials and letters were excluded from this study. The exclusion criteria were non-English journals, non-open access journals inaccessible through subscription, and articles dealing with other types of genu varum. Literature search was conducted on January 10, 2022 in PubMed, Science Direct, and ProQuest. Search keywords using Boolean logic were: *“osteotomy”* AND *“fixation”* AND *“Blount's disease”.* Any duplicate studies were first excluded, then study titles and abstracts were then screened further to eliminate unrelated articles passing the search filter. Only studies that met the PICOs criteria were reviewed. With P(patients): Patients with Blount disease; I(intervention) and C (Control): Osteotomy and Post-operative Fixations; O (Outcome): primary outcome is Functional outcome, the recovery or follow-up period, and/or the recurrence.

### 2.2 Study characteristics

This study reviewed osteotomy surgeries and fixation methods to correct deformities in Blount disease. The aim of this study is to enrich understanding about the recent advances of treatments for Blount disease. The population of the review is patients suffering from Blount disease. The interventions are pre-operative assessment and planning, different osteotomy surgeries and post-operative fixation methods. The outcomes include the patients’ self-assessment for functional outcome used different types of questionnaires, the recovery or follow-up period, and/or the recurrence.

### 2.3 Data analysis and quality assessment

Screening article was performed by two authors, Full-text analysis of selected articles was subsequently done based on previously set eligibility criteria. Then, necessary data were extracted from the selected journals to be further analyzed. Relevant data pooled from each study article were analyzed manually by using Microsoft Office 2019. The quality of studies was appraised using Oxford Centre for Evidence-Based Medicine (2011) Levels of Evidence. If there is disagreement at the final stage, we discuss with third authors by consensus for made decision for each eligibility article.

## 3 Results

### 3.1 Literature search

**Fig. 1:**
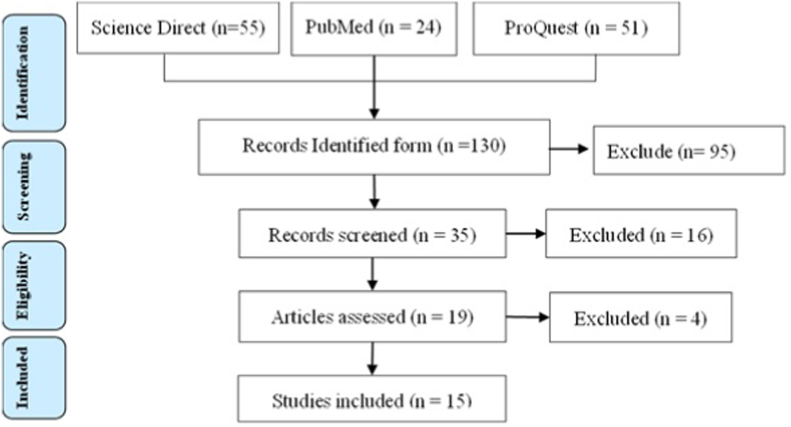
Literature searching process

Initial search with the keyword “*osteotomy”* AND “*fixation”* AND “*Blount's disease”.* We used three database PubMed, in this study Science Direct, and ProQuest. From initial searching yielded 24 articles in PubMed, 55 articles in Science Direct, and 51 article. From 130 article from initial searching, we exclude 106 articles because of 67 articles duplicates, 28 article ineligible by automation tools (English language and published within the last 10 years), and 16 studies were dropped after applying query syntax and article type criteria through tittle and abstract screening not matched with the study. Additional 4 studies were excluded because high not qualify regarding quality of studies by CEBM tools. Thus, leaving 15 articles included for review (Fig. 1).

### 3.2 Study characteristics

Of the 15 articles, seven of them are retrospective studies, four of them are case reports, and four are prospective studies. Two of the seven retrospective studies were comparison studies [7,8]. As many as 273 patients (114 males and 155 females) with 330 lower limb surgeries were included in the studies reviewed. Mean correction angle reported in the studies ranged from 17.64° [9] to 45° [5]. One case report was qualitative in nature and does not disclose the sex and age of the patient [10]. One report recounts the case of a 78-year-old patient after a 65-year follow-up [11]. The scope of this review is considered small but still covers various efforts to manage Blount diseases. Most prospective studies in this review provided information regarding the surgical planning through radiographic imaging, while most retrospective studies attempted to revisit patients’ wellbeing and self-assessment during the post-operative surgery and their subsequent follow-up (Table 1).

**Table 1:** Characteristics of studies.

First author	Year	Country	Type	Patients	# Tibia	Male	Female	Age (mean/range)
Gkiokas	2012	Greece	Retrospective	8	9	8	0	12 (9–14)
Karuppal	2016	India	Case	1	1	1	0	5
Edwards	2017	UK	Retrospective	7	8	1	6	9.5 (6.6–10.6)
Tersejen	2018	Croatia	Case	1	1	0	1	78
Abe	2018	Japan	Case	1	2	0	1	15
Griswold	2018	US	Retrospective	9	11	N/A	N/A	15
Miraj	2019	Indonesia	Prospective	18	27	12	6	7.8 (2–17)
Abraham	2019	US	Retrospective	23	29	9	20	9.9 (7–18)
Musikachart	2020	Thailand	Comparison	46	72	29	17	2.88 (2.17–3.92)
Cerqueira	2021	Brazil	Case	1	2	N/A	N/A	N/A
Ghasemi	2021	US	Comparison	79	79	0	79	44.3 (19–68)
Aly	2021	Egypt	Prospective	19	25	16	3	17.23 ± 5.27
Baraka	2021	Egypt	Prospective	19	21	6	13	10.3 (8.2–13.6)
Nada	2021	Egypt	Prospective	11	11	6	5	13.5 ± 1.1
Zein	2021	Egypt	Retrospective	30	32	26	4	16.6 (13–22)

The most featured technique featured in papers reviewed employed proximal tibial osteotomy [1,4,5,[7], [8], [9],^12^,^13^] with different location of surgical interventions, namely intra-epiphyseal [11], metaphyseal [[14], [15], [16]], and subperiosteal [17] sites. At least three procedures described were performed as double osteotomy in a single surgery [10,17,18], and at least two described grafting fibular fragments into tibial openings [17,18]. Various types of osteotomies were performed in the studies, the most prominent were the opening-wedge osteotomy [7,[9], [10], [11]] and dome-shaped osteotomy [8,17]. Three case studies were reporting novel techniques to manage unique cases requiring special procedures, such as controlled gradual opening [10], Z-shaped osteotomy [4], and inverted V-shaped osteotomy [13].

This review saw majority of the reported osteotomies were stabilized using internal fixations, with minimal screws [12], plates and screws [1,9,13,15,16], and K-wires [4,17,18]. The rest of external fixations described mainly used the hexapod rings resembling Ilizarov apparatus [5,14] and one considered using mono-lateral L-shaped pins to achieve gradual correction [10]. A patient with Turner syndrome (the absence of one X chromosome which cause various anatomic anomalies in the musculoskeletal system, including progressive varus deformity of the proximal tibia) was also reported to had undergone two bilateral osteotomy procedures with different types of fixation [13] (Table 2).

**Table 2:** Topics covered in the studies.

First author	Follow-up (mean/range)	Correction (degrees)	Fixation	Location	Type
Gkiokas	10 (5–15)	33	internal K-wires	double, grafted	wedge
Karuppal	2	22	internal K-wires	proximal	Z-angular
Edwards	4.6 (2.2–9)	31.88	external ring	metaphyseal hemiplateau	N/A
Tersejen	65	N/A	N/A	intraepiphyseal plateau	wedge (open)
Abe	1.5	23 and 32	internal plates	proximal	V-inverted
Griswold	1.45	17.64 (7–26)	internal plates	proximal	wedge (open)
Miraj	1	29	internal plates	proximal	V-step
Abraham	7.3 (2–22)	26	internal K-wires	subperiosteal, grafted	oblique fibula, dome tibia
Musikachart	4.77 ± 2.78	29.32 ± 7.98	N/A	proximal	64 dome 8 wedge
Cerqueira	N/A	qualitative	exolateral pins	double, gradual	wedge (open)
Ghasemi	N/A	various	43 internal 36 external	proximal	wedge (open)
Aly	3.4 (2–5)	22.08	internal screws	proximal	modified oblique
Baraka	5.1 (3.2–8.3)	36	internal plates	metaphyseal plateau	oblique-plane
Nada	2.2 ± 0.5	36.6 ± 5.1	internal plates	metaphyseal hemiplateau	wedge (close)
Zein	2.77 ± 0.56	25–45	external ring	proximal	N/A

## 4 Discussion

The clinical features of untreated Blount disease are progressive leg flexion and medial tibial torsion. Serial radiographs of the proximal tibia showed typical epiphyseal, growth plate and metaphyseal changes: decreased medial tibial epiphyseal height, increased downward slope, and irregular medial growth plate [17]. The mechanical regulation of the epiphysis is referred as the Hueter-Volkmann law, which states that the longitudinal growth plate of long bones is hindered by increased compressive forces on the posteromedial portion of the proximal tibia and stimulated by increased traction of the growth plates. This results in relative growth inhibition, which may be exacerbated by varus torque induced by gait patterns in obese patients. Associated deformities of the distal femur (varus or valgus) and distal tibia are also present [9].

In late-onset Blount disease, growth inhibition may lead to premature cessation of the medial epiphysis, which explains the success of unilateral valgus osteotomy in early-onset cases, but not in late-onset pediatric cases which require repeated osteotomy [18]. Nonoperative management of Blount disease may consider orthoses in patients younger than 3 years of age with mild unilateral disease. However, the effectiveness of brace therapy is controversial, with limited indications and difficult patient compliance. Therefore, even at an early stage, surgical intervention is considered the key to achieving permanent and lasting correction. Possible options for correcting the resulting angular deformity are epiphyseal fixation, osteotomy (acute or progressive correction), and progressive correction with distraction epiphyseal plates [10] (Table 3).

**Table 3:** Variables measured in featured studies.

Measurements	Auth. Abbr.	Abraham et al.	Aly et al.	Baraka et al.	Edwards et al.	Griswold et al.	Musikachart et al. [3]	Nada et al.
limb-length discrepancy	LLD			X				
deformity angle	DA						X	
anatomical axis	AA	X						
mechanical axis	MA	X	X	X	X	X	X	X
metaphyseal diaphyseal	MDA			X			X	
femoral-tibial angle (Q)	FTA		X	X			X	X
lateral distal femoral	LDFA	X		X	X	X		
medial proximal tibial	MPTA		X	X	X	X	X	
medial plateau	MPA			X	X			X
medial tibial	MTA	X	X					
posterior proximal tibial	PPTA		X	X			X	
posterior medial tibial	PMTA	X						
Total variables measured		5	5	8	4	3	6	3

Blount disease management should be personalized based on age, classification, severity of deformity, differences in limb length, and the experience of the surgeons. The goal of Blount's disease treatment is to obtain a lower extremity with normal joint heritability and orientation, as well as the same length in both extremities when skeletal maturity is reached. Generally, children 2–5 years of age are treated with observation or trial braces, while late or progressive deformities are treated with surgery [1]. Abnormal lower limb biomechanical axis causes damage to knee cartilage and affects the result of knee ligament reconstruction surgery. Inaccurate correction of varus deformation could lead to persistent pain [7] and cause socio-psychological problems like struggling with schooling, discrimination, low self-esteem, and depression for the patient or family [8].

### 4.1 Pre-operative screening and surgical planning

The first step of surgical planning is the identification of center of rotation of angulation (CORA). Blount disease consists of a multi-apical deformity, which means there are two CORAs: one standing at the epiphyseal plate and one at metaphyseal level [10]. Preoperative radiography was used to calculate the correction of deformities and Langenskiöld staging system was used to assess the severity. Often, various terms were used to describe the same variable throughout studies. There is a need to standardize abbreviation across orthopedic research texts in order to improve consistency.

Analysis of pre-operative radiological evaluation was based on standing anteroposterior (AP) and frontal and lateral views of both lower limbs. Seven non-case report studies measured pre-operative and post-operative mechanical axis (MA) angle. The degree of correction was calculated by subtracting the MA at the latest follow-up from the preoperative MA [9]. Three most recorded variable to determine the surgical success following MA were medial proximal tibial angle (MPTA), femoral-tibial angle (FTA), and lateral distal femoral angle (LDFA), respectively. All featured studies reviewed had different methods to assess the deformity and determine important variables that needs to be addressed during the pre-operative screening and post-operative follow-up. First, different imaging techniques used to take the radiographs might not always have been standardized, so real measurements of the radiographic angles were often subjected to individual perception [14,17]. Second, the chronicity and severity of the medial tibial plateau deformation in children and adolescents made the comparison of some radiographic measurements unable to be translated into the typical standard values in adults [17]. Plain 2D radiographic reading has the tendency to overestimate the apparent depression of the medial plateau, and the use of an intra-operative arthrogram was proposed to provide information on the two-plane geometry of the articular correction [14].

### 4.2 Osteotomy procedures and types

Proximal tibial osteotomy, or sometimes referred to as high tibia osteotomy, is a proven surgical method which attempts to realign lower limb mechanical axis to correct knee problems, initially to slow the progression of osteoarthritis. In Blount disease, the load is transferred to the medial compartment, resulting to knee deformation. Proximal tibial osteotomy coupled with mono-lateral external frame is a technique where the fibula and lateral tibial cortex are left intact during osteotomy. Realignment hinges on the gradual distraction of the medial cortex, through the intact lateral cortex [7]. Even severe tibial deformities associated with late presenting Blount disease could mostly be treated successfully by double PTO done within a single procedure [17].

Different surgeries have been described to correct angular and rotational problems, such as open-wedge, close-wedge, oblique, and dome osteotomies [16]. Closing-wedge osteotomy might cause additional limb shortening [4,15] but does not require iliac crest grafting in contrast to opening-wedge and the lower bony block securely supports the medial plateau elevation [15]. Opening-wedge osteotomy does not allow the correction of rotational deformities or limb length discrepancy [9], and might cause under-correction of the internal tibial torsion and site instability which requires rigid internal fixation [4]. There was no statistically significant difference in tibial slope between the outcomes of dome and wedge-shaped proximal tibial osteotomy, with a particular focus on sagittal alignment of the knee joint at any measured time interval [8].

Aly et al. proposed a modified Rab osteotomy (single oblique proximal tibial osteotomy). In this procedure, the rapid union of the two bone fragments simultaneously remodeled the internal torsion of the axial plane, varus deformity along the coronal plane, and procurvatum in the sagittal plane. This procedure is not preferable if there is a medial condyle depression or leg-length discrepancy [12]. Two stage ‘inside-out’ oblique-plane osteotomy from Baraka et al. has been reported to preserve articular hinge, allowing for precise joint-levelling, and to correct varus and internal tibial torsion. This single-stage double-grafting osteotomy was done while avoiding the potential risk of intra-articular fractures and medial condyle displacement, preventing iatrogenic translational deformities previously noted with other types of osteotomies [16].

Inverted V-shaped PTO was able to correct severe bilateral tibia vara which had recurred after corrective proximal tibial osteotomy below the growth plates [13]. Miraj et al. also performed a step cut V-shaped tibial osteotomy inspired by a modification of step cut osteotomy for cubitus varus deformity [1]. The Z-shaped osteotomy reported by Karuppal et al. is a modification of wedge-shaped osteotomies to address special geometric challenges, in which rotational deformity can be simultaneously corrected without compromising bone stability and contour [4]. Some of these experimental procedures are reportedly advantageous in personalized cases, providing enough correction with large bony stock and wider contact [1,4,13,16]. It requires smaller amounts of resection and opening when compared to both wedge-shaped osteotomies and also help preserve patellar height where other types of osteotomies failed [13,15,16].

### 4.3 Corrective fixation techniques

Many post-operative fixation methods to aid correction and healing have also been developed, from calcified cast immobilization, Kirschner-wires, staples and screws, locking compression plates, to external fixators like mono-lateral L-shaped lock, Ilizarov apparatus, and Taylor spatial frames (TSF) [7,16]. In both fixation methods, pin breakage and pin site infections around screws or wires were the most frequent complication encountered [12,16], however deep infections requiring a complete debridement was a rare occurrence. Multiple drilling holes and screw tampering can cause loosening and infections, especially in the proximal metaphyseal part [5].

Internal locking compression plates secures both the metaphyseal oblique osteotomies and medial plateau elevation, thus providing support to the medial condyle elevated to the surface, meanwhile screws securely traverse the epiphysis to ensure lateral epiphysiodesis [16]. Internal fixation methods did not offer rigidity and able to preclude the use of plaster immobilization [16]. Patients with internal fixation are able to begin knee range movements earlier, but with the risk of medial condyle displacement, loss of correction, and revision surgeries [16].

Circular external fixators guarantee more security over mono-axial external fixator as screws can be applied and adjusted through multiple different planes to achieve better fixation following osteotomy [5,17]. They also allow early weight-bearing [15] and address limb-length discrepancy in obese patients [12]. The main drawback of gradual mono-axial external fixator is the cumbersome use of the locking mechanism to perform gradual correction four times a day. The patient undergone this alignment surgery should be properly trained to perform device rotation with the L-shaped key [10]. Adding weight to the extra burden of the daily care of the external devices, internal fixation option is still more appealing in some cases [15].

For severe deformities, the follow-up of the gradual correction of tibial osteotomy and plateau elevation was required at longer periods for radiographic imaging and detection of callus [10]. Gradual correction using external fixator could tune correction post-operatively [15], but its profound disadvantage is the prolonged duration of recovery [13]. Evaluation of the changes in the mechanical axis deviation in both external and internal fixation techniques showed no significant difference in the accuracy of deformity correction. However, the patellar tendon height changed smaller in external fixation group than that in internal fixation group [7].

### 4.4 Post-operative complications and follow-up

Besides at least four cases of partial common peroneal nerve paresthesia [14,17] and lateral tingling sensation in one leg [12], the complications resulting from acute correctional osteotomies were quite low. There were zero cases of septic arthritis, osteomyelitis, vascular necrosis, misalignment, or union failure reported by both Abraham et al. and Zein et al. [5,17] and none of the patients observed by three other similar studies had post-operative compartment syndrome or neurological problems [1,5,9]. Karuppal et al. also reported no major complications or any neurological problems after his novel Z-shaped osteotomy [4]. The only common problem across all studies were surgical site infections and could be managed by several courses of oral antibiotics and proper wound care.

Edwards et al. outlined that two patients (three limbs) who had recurrences with persistent torsional asymmetry were caused by the failure to appreciate the extent of the pre-existing deformity during pre-operative surgical planning [14]. Baraka et al. reported no hardware failure until three to four years of internal plate removal, but two patients developed hypertrophic scar on surgical sites [16]. Through linear regression analysis, it was estimated that every 1 kg/m^2^ increase of BMI leads to longer healing period by 1.1165 days [12]. There is a unique follow-up of a 78-year-old patient who undergone radiographic examination 65 years after her three surgeries (aged 8, 12, and 13) between two relapses [11]. She underwent her final intra-epiphyseal osteotomy of the medial tibial condyle with elevation of the medial tibial plateau in 1951. She had good gait function, and no pain on usual activities like skiing, biking, and swimming.

### 4.5 Subjective assessment and future recurrences

Studies used slightly distinctive scales to evaluate patients' pre-operative condition, post-operative well-being, and their subsequent follow-ups. The most used type of qualitative scale is derived from Pediatric Outcomes Data Collection Instrument (PODCI) developed mainly in the United States institutions [15,16,18]. A 20-item questionnaire based on PODCI consisting of four categories (general satisfaction, mobility, sports activity, happiness) was used by Gkiokas et al. to rate patients' satisfaction and opinion regarding their quality of life [18]. Abraham et al. used a 93-point scale and recorded the questionnaire scores into percentiles: excellent, good, and fair scores [17]. Unrelated questions like perceived upper limb functions were often excluded from the original PODCI scale [15]. Cosmetic appearance, comfort, stability during long-distance walking and short-distance running were the main improvements that impacted patients’ satisfaction in all studies featuring modified PODCI subjective scales [15,17,18]. One qualitative measurement by Aly et al. functionally assessed the outcomes using Lysholm knee scoring scale, consisting of eight main items: limp, support, locking, instability, pain, swelling, stair climbing, and squatting [12].

Factors influencing the recurrence rates of abnormal medial articular slope are the failure to impede lateral plate growth, improper 3D angle correction, older age during surgical treatment, large tibial-femoral or Drennan angle, and higher Langenskiöld classification types [1,17]. Recurrence usually occurs if osteotomies were performed on patients aged 8 years and later [11]. Miraj et al. concurred that recurrence rate was strongly correlated with the patients’ age undergoing the surgical procedure, even as early as 4 years or age [1]. Even with early management, it is reported that deformities often recurs when tibial valgus osteotomy is not adequately corrected, and the elevation of tibial plateau must be performed [15,18]. Edwards et al. proved that failure of full correction is caused by the attempt to simultaneously correct of all variables [14]. Overcorrection has been a habitual practice by pediatric orthopedic surgeons to prevent recurrence [5], but results in worse cosmetic appearance and weird gait [15].

This study also has several limitations, there is heterogeneity from data sources. Therefore, so the specific conclusion is still not sufficiency. Limited access to full journals will also affect the results of the search and interpretation. Several questions remain unanswered at present. A further study with more focus on randomized control trial study therefore suggested.

## 5 Conclusion

Blount disease represents a large spectrum of pathology with a common mechanical pathogenesis leading to medial tibial growth suppression and deformity. Projected limb length discrepancy must be calculated and addressed. Surgical options depend on the patient's age, extent of physeal involvement, severity, and the number of deformities. Despite the unpredictable results and frequent failure, most authors recommend corrective osteotomy preferably before age 4 with options for acute or gradual correction depending on the patient's age, BMI, correction angle, and complexity of deformity. The goals of surgery are to restore normal joint and limb alignment, equalize limb lengths at skeletal maturity, and prevent recurrence. Completion of lateral tibia and proximal fibula epiphysiodesis by means of internal or external fixators showed no significant difference and should be tailored into individual cases.

## Ethical approval

This study not need Ethical Review.

## Sources of funding

This study did not receive any specific grant from funding agencies, commercial, or not-for-profit sectors.

## Author statement

Panji Sananta: conceptualization, writing original draft preparation, supervision.

Joko Santoso: writing the paper and editing, data interpretation, data collecting.

Muhammad Alwy Sugiarto: writing the paper and editing, data interpretation.

## Trail registry number

1. Name of the registry: PROSPERO
2. Unique Identifying number or registration ID: 316258
3. Hyperlink to your specific registration (must be publicly accessible and will be checked):

## Guarantor

Panji Sananta.

Orthopedic and Traumatology Department, Faculty of Medicine, Universitas Brawijaya-Saiful Anwar General Hospital.

Jl. Jaksa Agung Suprapto No.2, Klojen, Malang 65112, East Java, Indonesia.

E-mail address: panjisananta@ub.ac.id.

## Annals of medicine and surgery

The following information is required for submission. Please note that failure to respond to these questions/statements will mean your submission will be returned. If you have nothing to declare in any of these categories then this should be stated.

## Consent

Studies on patients or volunteers require ethics committee approval and fully informed written consent which should be documented in the paper.

Authors must obtain written and signed consent to publish a case report from the patient (or, where applicable, the patient's guardian or next of kin) prior to submission. We ask Authors to confirm as part of the submission process that such consent has been obtained, and the manuscript must include a statement to this effect in a consent section at the end of the manuscript, as follows: “Written informed consent was obtained from the patient for publication of this case report and accompanying images. A copy of the written consent is available for review by the Editor-in-Chief of this journal on request”.

Patients have a right to privacy. Patients’ and volunteers' names, initials, or hospital numbers should not be used. Images of patients or volunteers should not be used unless the information is essential for scientific purposes and explicit permission has been given as part of the consent. If such consent is made subject to any conditions, the Editor in Chief must be made aware of all such conditions.

Even where consent has been given, identifying details should be omitted if they are not essential. If identifying characteristics are altered to protect anonymity, such as in genetic pedigrees, authors should provide assurance that alterations do not distort scientific meaning and editors should so note.

This study doesn't need inform consent.

## Provenance and peer review

Not commissioned, externally peer-reviewed.

## Declaration of competing interest

We declare that they have no known competing financial interests or personal relationships that could have appeared to influence the work reported in this paper.
